# Supplementary material for: Dietary Behaviour Is Associated with Cardiometabolic and Psychological Risk Indicators in Female Hospital Nurses—A Post-Hoc, Cross-Sectional Study
Source: Nutrients. 2019 Sep 2;11(9):2054. doi: 10.3390/nu11092054 (PMC6770286; doi:10.3390/nu11092054)
Supplement: Supplementary file 1 [file nutrients-11-02054-s001.pdf]

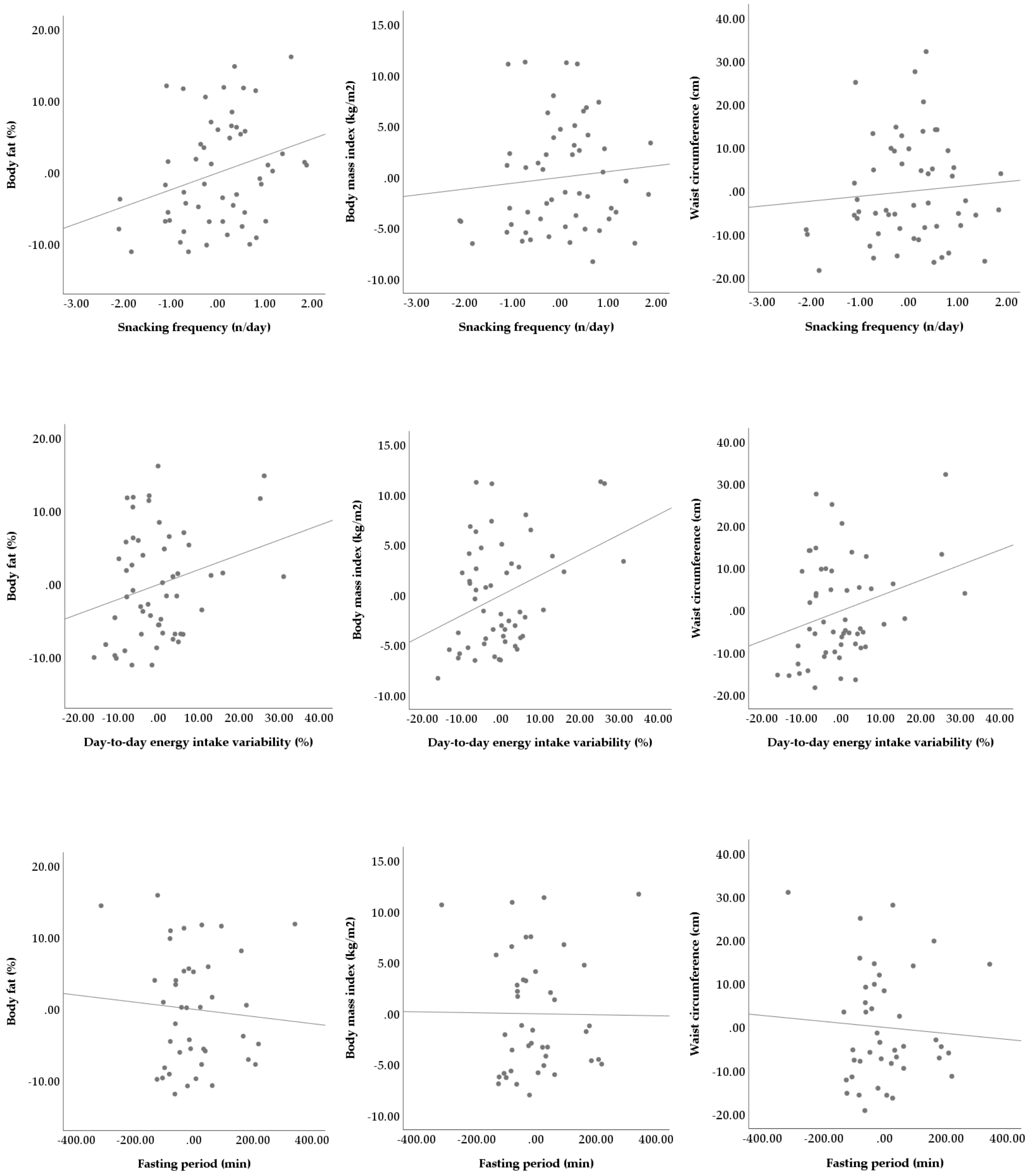

Figure S1. Correlations between dietary behaviours and anthropometric measures. Age and daily caloric intake adjusted data are presented.
